# Genome survey of *Zanthoxylum bungeanum* and development of genomic-SSR markers in congeneric species

**DOI:** 10.1042/BSR20201101

**Published:** 2020-06-26

**Authors:** Jingmiao Li, Siqiao Li, Lijuan Kong, Lihua Wang, Anzhi Wei, Yulin Liu

**Affiliations:** 1 College of Forestry, Northwest A&F University, Yangling 712100, China; 2 School of Life Sciences, Yunnan University, Kunming 650500, China; 3 Institute of Biotechnology and Seed, Sichuan Academy of Forestry Science, Chengdu 610081, China

**Keywords:** Genetic diversity, Genome size, Genome survey, SSR markers, Zanthoxylum bungeanum

## Abstract

*Zanthoxylum bungeanum*, a spice and medicinal plant, is cultivated in many parts of China and some countries in Southeast Asia; however, data on its genome are lacking. In the present study, we performed a whole-genome survey and developed novel genomic-SSR markers of *Z. bungeanum*. Clean data (∼197.16 Gb) were obtained and assembled into 11185221 scaffolds with an N50 of 183 bp. *K*-mer analysis revealed that *Z. bungeanum* has an estimated genome size of 3971.92 Mb, and the GC content, heterozygous rate, and repeat sequence rate are 37.21%, 1.73%, and 86.04%, respectively. These results indicate that the genome of *Z. bungeanum* is complex. Furthermore, 27153 simple sequence repeat (SSR) loci were identified from 57288 scaffolds with a minimum length > 1 kb. Mononucleotide repeats (19706) were the most abundant type, followed by dinucleotide repeats (5154). The most common motifs were A/T, followed by AT/AT; these SSRs accounted for 71.42% and 11.84% of all repeats, respectively. A total of 21243 non-repeating primer pairs were designed, and 100 were randomly selected and validated by PCR analysis using DNA from 10 *Z. bungeanum* individuals and 5 *Zanthoxylum armatum* individuals. Finally, 36 polymorphic SSR markers were developed with polymorphism information content (PIC) values ranging from 0.16 to 0.75. Cluster analysis revealed that Z. *bungeanum* and *Z. armatum* could be divided into two major clusters, suggesting that these newly developed SSR markers are useful for genetic diversity and germplasm resource identification in *Z. bungeanum* and *Z. armatum*.

## Introduction

**Figure 1:**
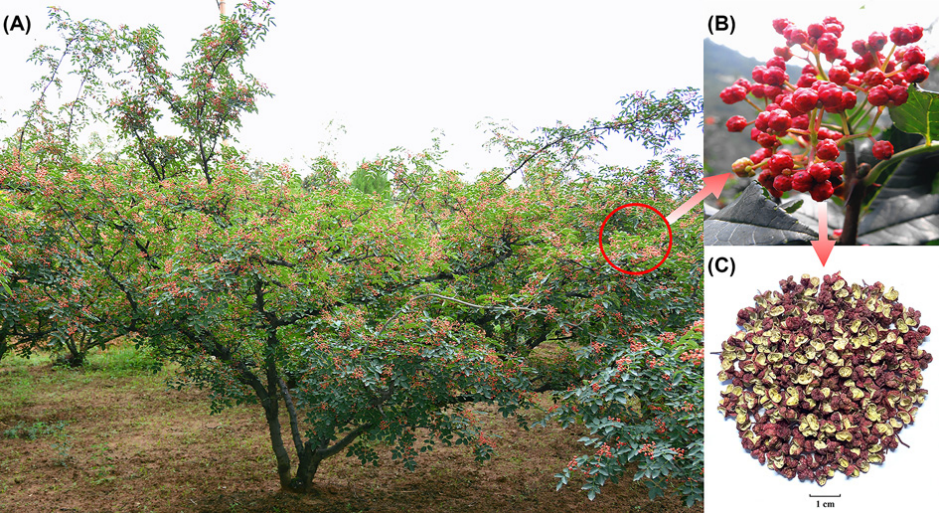
The adult tree, fruits and dried pericarps of ZB (**A**) An adult tree covered with ripe fruits. (**B**) The appearance of the fruits. (**C**) Characteristic of dried pericarps.

The genus *Zanthoxylum*, in the family Rutaceae, consists of approximately 250 species and is distributed worldwide [1]. *Zanthoxylum bungeanum* (ZB), also referred to as ‘Chinese prickly ash’, ‘Sichuan pepper’ is a representative species of *Zanthoxylum* in China and Southeast Asia (Figure 1). The pericarps of ZB have been widely used as a traditional culinary spice and Chinese herbal medicine for thousands of years due to its flavor and medicinal characteristics [2]. As an economically important species, the cultivation area of ZB and a closely related species, *Zanthoxylum armatum* (ZA), occupies approximately 1.67 million hectares and produces an annual output of 350000 tons of dried pericarp. The economic value of this agricultural industry generates more than 4 billion US dollars in China. This level of economic value has generated interest in increasing the molecular data available for ZB. Although some transcriptome information has been obtained from several tissues in ZB [3,4], data about the genome structure of ZB are lacking.

Because of recent advances in DNA sequencing techniques, draft genomes have been assembled for many plant species as resources for genomic and genetic research efforts [5–7]. However, the genomes of some plant species, particularly tree species, are highly heterozygous, have a complex genetic background, and have an unknown genome size. Therefore, before large-scale sequencing is initiated, basic biological characteristics of the target material are evaluated, such as chromosome ploidy analysis or low-coverage genome sequencing (also known as a genome survey), to gauge the complexity of the genome and provide a reference for whole-genome sequencing [8].

Genome surveys, which use next-generation sequencing (NGS), yield a large amount of genomic data in a rapid, cost-effective manner. Genomic data from genome surveys not only provide useful information on genome structure, such as an estimation of genome size, heterozygosity levels, and repeat contents but also establish a genomic sequence resource from which molecular markers can be developed [9–11].

Simple sequence repeats (SSRs), also referred to as microsatellites or short tandem repeats (STRs) are tandem repeats of 1–6 nucleotides that are widely distributed in eukaryotic genomes [12]. Depending on the location of an SSR, it can be classified as either a genomic-SSR (G-SSR) or an expressed sequence tag (EST)-SSR, which indicates whether the SSR is either in a non-coding region or a translated region, respectively [13]. SSR markers are used for genetic and evolutionary analysis, germplasm resource identification, genetic map construction, and marker-assisted selective breeding because of their wide distribution, high polymorphism, and co-dominance and because the cost of developing these markers is low [14]. Therefore, increasing the number of SSR markers for ZB will provide a useful resource for genetic research.

In the present study, the main objectives were (1) to obtain information about the genome size, GC content, repeat sequence rate and heterozygosity rate of ZB by a genome survey; (2) to identify SSRs in assembled genomic sequences of ZB; and (3) to develop and evaluate G-SSR markers to assess genetic diversity in ZB and ZA (a plant that is used in the same manner as ZB in Southwest China).

## Materials and methods

### Plant materials and DNA extraction

Healthy leaves were collected from one ZB adult plant from the Yangling comprehensive test and demonstration station of Northwest A&F University in Shaanxi, China. The leaves were cleaned with purified water, immersed in liquid nitrogen, and stored at −80°C until use. For polymorphic marker screening, ten ZB individuals (ZB01–ZB10) and five ZA individuals (ZA01–ZA05) were collected from six provinces in China (Table 1). All samples were collected as young leaves from three individual plants that were combined for DNA isolation. Genomic DNA was extracted using a plant DNA extraction kit (DP305; Tiangen, Beijing, China), and the quality and quantity of the isolated DNA were evaluated by 1% agarose gel electrophoresis and NanoPhotometer spectrophotometer (Implen NanoPhotometer, Westlake Village, CA, U.S.A.).

**Table 1:** Origin regions of 15 *Zanthoxylum* individuals

Species	ID	Individual name	Origin region
*Z. armatum*	ZA01	Chongqingjiuyeqing	Jiangjin, Chongqing, China
	ZA02	Pengxiqinghuajiao	Suining, Sichuan, China
	ZA03	Rongchangwuci	Rongchang, Chongqing, China
	ZA04	Hanyuanputaoqingjiao	Ya’an, Sichuan, China
	ZA05	Goujiao	Ya’an, Sichuan, China
*Z. bungeanum*	ZB01	Youhuajiao	Liupanshu, Guizhou, China
	ZB02	Suhuajiao	Liupanshu, Guizhou, China
	ZB03	Hanyuandahongpao	Ya’an, Sichuan, China
	ZB04	Shandongdahongpao	Linyi, Shandong, China
	ZB05	Shanxidahongpao	Yongji, Shanxi, China
	ZB06	Fengxiandahongpao	Baoji, Shaanxi, China
	ZB07	Fuguhuajiao	Yulin, Shaanxi, China
	ZB08	Shizitou	Hancheng, Shaanxi, China
	ZB09	Hanchengdahongpao	Hancheng, Shaanxi, China
	ZB10	Dangcunwuci	Hancheng, Shaanxi, China

### Genome survey sequencing, assembly, and estimation of genomic characteristics

Genomic DNA samples were randomly sheared into two collections of average fragment size (250 or 350 bp) using ultrasound (Covaris, U.S.A.) and used to construct four libraries (two libraries from each fragment collection) for sequencing. Library construction and sequencing were performed by Beijing Novogene Biological Information Technology, Beijing, China (http://www.novogene.com/) using an Illumina HiSeq 2000 platform. According to the genome size of *Zanthoxylum oxyphyllum* (3.7Gb) [15], we estimated that the genome size of ZB was approximately 4 Gb. Consequently, to ensure at least 50× coverage of the ZB genome, 238.5 Gb raw data were generated. After filtering to remove adaptors, poly(N) sequences, and low-quality reads, the remaining clean reads were used for *K*-mer analysis. Based on the results of the *K*-mer analysis, 17-mers (*K* = 17) were used to estimate the genomic characteristics, including genome size, repeat sequence rate, and heterozygosity rate. Furthermore, clean data were assembled (*K* = 41) into contigs and scaffolds using the De Bruijn graph-based assembler, SOAPdenovo (Version 1.05, BGI, Beijing, China) [16]. The GC content was calculated with contigs longer than 500 bp. More details regarding the analysis procedures employed in this study have been described by Bi et al. [17].

### SSR identification, primer design, and polymorphism screening

SSR loci were detected from scaffolds longer than 1000 bp using MISA software (Microsatellite, http://pgrc.ipk-gatersleben.de/misa/). For mono-, di-, tri-, tetra-, penta-, and hexanucleotide SSRs, the search parameters were set with minimum repeat numbers of 10, 6, 5, 5, 5, and 5, respectively. Primers were designed using Primer 3.0 software (https://primer3.sourceforge.net/webif.php), following the following parameters: 100–300 bp for amplification length, 18–27 bp for primer size, 40–70% for primer GC content, and 57–63°C for melting temperature. The PCR reaction volume was 20 μl and consisted of 10 μl 2× Taq Master Mix (Vazyme, Nanjing, China), 5 μl genomic DNA (20 ng/μl), 1.5 μl (2 μmol/l) each forward and reverse primers and 2 μl ddH_2_O. The PCR program was 5 min at 95°C; followed by 35 cycles of 95°C for 30 s, 55°C for 40 s, and 70°C for 40 s; and a final extension of 72°C for 8 min. The PCR products were separated by electrophoresis on an 8% denaturing polyacrylamide gel, visualized by silver staining, and fragment sizes were estimated using pBR322 DNA/*Msp*I markers (Tiangen, Beijing, China).

### Data analysis

Genetic diversity parameters, such as the number of alleles (*N*_a_), observed heterozygosity (Ho), and expected heterozygosity (He) were calculated using POPGENE1.32 [18], and the polymorphism information content (PIC) values were calculated using the formula $\text{PIC}=1-\Sigma \;{\text{f}}_{i}^{2}$, where *f_i_* is the frequency of the *i-*th allele [19]. The dendrogram of 15 individuals was constructed by UPGMA clustering using NTSYSpc2.10 software to reveal genetic relationships [20].

## Results and discussion

### Genome sequencing and *K*-mer analysis

In the present study, 238.5 Gb of raw sequence data were generated by four small-insert libraries. After removing low-quality reads, 197.16 Gb of clean data were used for the *K*-mer analysis. In the four small-insert libraries, the Q20, Q30, and GC contents were 97.28–98.10%, 93.88–95.66%, and 38.38–38.68%, respectively. Moreover, the error rate was 0.02% for each library (Table 2). With an Illumina platform, the overall accuracy of the sequencing is indicated by having Q20 and Q30 values of at least 90% and 85%, respectively [21]. Therefore, the sequencing accuracy of the ZB genome survey in the present study was high.

**Table 2:** Basic statistics for the genome survey sequencing data of ZB

Library	DES00802	DES00803	DES00804	DES00805
Insert size(bp)	250	250	350	350
Raw reads	200690359	210996601	192712146	190585631
Raw base(bp)	60207107700	63298980300	57813643800	57175689300
Effective rate (%)	76.70	76.24	89.43	89.43
Clean base(bp)	46124187900	48205998000	51133569600	51701329800
Error rate(%)	0.02	0.02	0.02	0.02
Q20(%)	98.10	98.07	97.28	97.40
Q30(%)	95.66	95.42	93.88	94.11
GC content(%)	38.66	38.68	38.38	38.39

**Figure 2:**
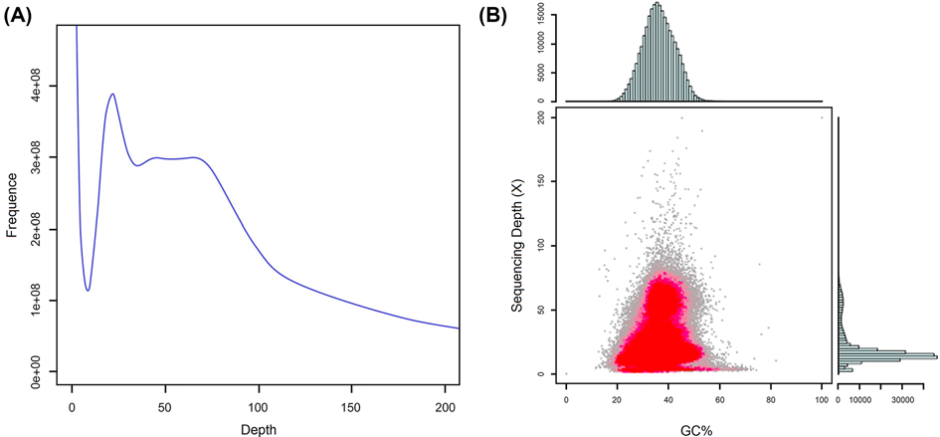
Distribution of *K*-mer = 17 depth and GC content and depth correlation analysis (**A**) In the figure, the estimated genome size of ZB was judged by the following formula: genome size = *K*-mer number/*K*-mer depth. The *x*-axis is depth; the *y*-axis represents the frequency at a particular depth divided by the total frequency of all depths. (**B**) In the figure, the *x*-axis represents the GC content, the *y*-axis represents the sequencing depth. The right side is the sequencing depth distribution and the top side is the GC content distribution. The red part represents the dense part of the points in the scatter plot.

In the 17-mer frequency distribution, the *K*-mer number was 176134142868, and the *K*-mer depth was 44. Based on the empirical formula (genome size = *K*-mer number/*K*-mer depth), the initial estimate of genome size of ZB is 4003.05 Mb. After excluding the effects of erroneous *K*-mers, the revised genome size is 3971.92 Mb (Table 3). Furthermore, based on the *K*-mer map, a high peak (22) was observed at half the *K*-mer depth (44), which indicates that the ZB genome has high heterozygosity, and the heterozygosity rate is estimated to be 1.73%. In addition, a fat tail was observed in the *K*-mer analysis, and the repeat sequence rate was calculated to be 86.04% (Figure 2A). The heterozygosity and repeat sequence rates of the ZB genome are much higher than those reported from the genome survey data of other woody plants, such as *Acer truncatum* (1.06%; 48.80%) [8], *Xanthoceras sorbifolium* (0.89%; 62.00%) [17], and *Betula platyphylla* (1.22%; 62.20%) [22]. Because of the high values for heterozygosity and repeat sequence rates combined with the chromosome number of ZB (2*n* = 136) [23], we speculate that ZB has a very complex genome.

**Table 3:** Estimation statistics and analysis based on *K*-mer of ZB

*K*-mer	*K*-mer number	K-mer depth	Genome size (Mb)	Revised genome size (Mb)	Heterozygous ratio (%)	Repeat (%)
17	176134142868	44	4003.05	3971.92	1.73	86.04

### De novo assembly and GC content analysis

Preliminary genome assembly was performed using clean reads. The software SOAPdenovo generated 11185221 contigs and 11086925 scaffolds using a *K*-mer length of 41. The maximum length and N50 length of contigs are 13151 and 183 bp, respectively, and for scaffolds, these values are 13628 and 186 bp, respectively (Table 4). Although a large amount of clean data (197.16 Gb) were used for assembly, the assembly results were unsatisfactory. The N50 lengths of contigs and scaffolds are notably shorter than those calculated in other similar studies [8,17,22]. A likely reason for these findings is that the ZB genome contains 68 chromosomes (2*n* = 136), has a high heterozygosity rate, and has a large number of repeated sequences; furthermore, the insert sizes for the sequencing libraries are relatively short (250 and 350 bp). Collectively, these factors likely contributed to the unsatisfactory assembly results.

**Table 4:** Statistics of the assembled genome sequences in ZB

	Total length (bp)	Total number	Total number(>2 kb)	Max length (bp)	N50 length (bp)	N90 length (bp)
Contig	2069338941	11185221	7712	13151	183	110
Scaffold	2072641802	11086925	7921	13628	186	111

GC content analysis was performed with contigs longer than 500 bp. Figure 2B shows the relationship between GC content and sequencing depth. The data suggest that the GC content of the ZB genome is approximately 37.21%, which is higher than the GC content in *Citrus* plants (32.0–35.0%) within the same family (Rutaceae) as ZB [24,25]. A scatter plot of GC content shows that the data segregate into two layers, a result that is likely due to the high heterozygosity rate (1.73%) [11]. GC content can influence the quality of genome sequencing. Because different densities of GC content can reduce the sequencing coverage in certain genomic regions, sequencing bias can occur on the Illumina sequencing platform and affect the genome assembly [26,27]. However, when GC content is between 30% and 50%, there is no significant influence on genome sequence quality [28]. Consequently, the 37.21% GC content of the ZB genome is not likely to have influenced the assembly results in the present study.

Based on the complex characteristics of the ZB genome, we recommend using second-generation sequencing (Illumina) combined with third-generation sequencing (PacBio) and using the Hi-C technique and BioNano Genomics for supplement in future whole-genome sequencing studies.

### SSR loci identification and primer design

Assembled scaffolds (57288) with a minimum length and total length of 1000 bp and 86.51 Mb, respectively, were selected for SSR searching by MISA software, and 27153 putative SSRs were identified on 17593 scaffolds. The mean distance (3.19 kb) between G-SSR loci in the ZB genome was longer than mean distances measured in *Ziziphus jujuba* (0.93 kb) [29], *Dioscorea zingiberensis* (1.94 kb) [30], and *Saccharina japonica* (2.2 kb) [31]. One possible reason is that the scaffolds we analyzed only account for 2.18% of the total size of the ZB genome.

Mononucleotides are the most abundant type of SSR and account for 72.57% (19706) of the total. Dinucleotides are the second-most abundant type of SSR (18.98%, 5154), followed by trinucleotides (6.85%, 1860), and tetra-nucleotides (1.14%, 309). Pentanucleotides and hexanucleotides SSRs are found at 60 and 64 loci, which account for 0.22% and 0.24% of the total, respectively. As the repeat motif length increases, the number of SSR loci decreases. Among mononucleotides, A/T motifs are the predominant type (98.41%). Among dinucleotides, the most frequent motifs are AT/AT (62.36%), followed by AG/CT (19.95%) and AC/GT (17.56%); however, CG/CG accounts for just 0.13%. Among trinucleotides, the most frequent motif is AAT/ATT (56.53%), followed by AAG/CTT (20.82%), and ATC/GAT (7.92%). ACG/CGT motifs are the least frequent, with only 16 loci (0.89%). Moreover, the most abundant motifs among tetra-, penta- and hexanucleotides are AAAT/ATTT (51.46%), AAAAT/ATTTT (45.00%), and AAAAAT/ATTTTT (15.63), respectively. According to these results, motifs containing only A and T residues are more common than those containing at least one C or G base, especially among di- and trinucleotides (Table 5).

**Table 5:** Distribution pattern of G-SSR motifs in 17593 scaffolds in ZB

Repeat motif	Number of repeats								Total
	5	6	7	8	9	10	10-20	>20	
Mono-nucleotide (19706)									
A/T						8006	10600	787	19393
C/G						32	204	77	313
Di-nucleotide (5154)									
AT/AT		897	540	444	326	285	720	2	3214
AG/CT		498	220	136	72	37	64	1	1028
AC/GT		416	186	107	75	37	81	3	905
CG/CG		4	3						7
Tri-nucleotide (1806)									
AAT/ATT	451	206	124	68	49	43	79	1	1021
AAG/CTT	201	82	36	13	19	7	17	1	376
ATC/GAT	81	32	8	8	2	4	8		143
AAC/GTT	42	23	9	5		1	3		83
ACC/GGT	52	13	7	3	1				76
AGG/CCT	33	18	4	4	3	1			63
AGC/GCT	36	5	3	2					46
CCG/CGG	9	5	2	1	1				18
ACT/AGT	11	2	2				3		18
ACG/CGT	9	2	3	2					16
Tetra-nucleotide (309)									
AAAT/ATTT	130	21	6	2					159
ACAT/ATGT	22	7	3	5	6				43
AAAG/CTTT	26	3	2		1				32
AATT/AATT	28	2	2						32
AAAC/GTTT	20	2	1						23
Others	13	4	2		1				20
Penta-nucleotide (60)									
AAAAT/ATTTT	21	6							27
AAAAC/GTTTT	8								8
AAATT/AATTT	3	1							4
AATCG/ATTCG	4								4
Others	14	2		1					17
Hexa-nucleotide (64)									
AAAAAT/ATTTTT	9	1							10
AAAAAC/GTTTTT	3	3							6
AAATAT/ATATTT	2	2							4
AAAAAG/CTTTTT	2	1							3
Others	32	8	1						41
Total	1262	2266	1164	801	556	8453	11779	872	27153

There is a certain relationship between the diversity of SSRs and motif sequence types. Because the energy required to destabilize the two hydrogen bonds between A and T is lower than that required to destabilize the three hydrogen bonds between G and C, the slippage rate of A/T is higher than G/C between the two DNA strands. The elevated slippage rate causes A/T to be more frequently observed in SSR motifs [32]. Other possibilities include the insertion of 3′-terminal poly(A) sequences into the genome or the conversion of methylated C residues to T residues [33].

To identify SSR loci that could have utility as potential markers, 21243 non-redundant primer pairs for 23475 G-SSR loci were designed using Primer 3 software (Supplementary Table S1); the remaining loci may have had flanking sequences that were too short or inappropriate for primer design.

### Genomic SSR marker development and cluster analysis

**Figure 3:**
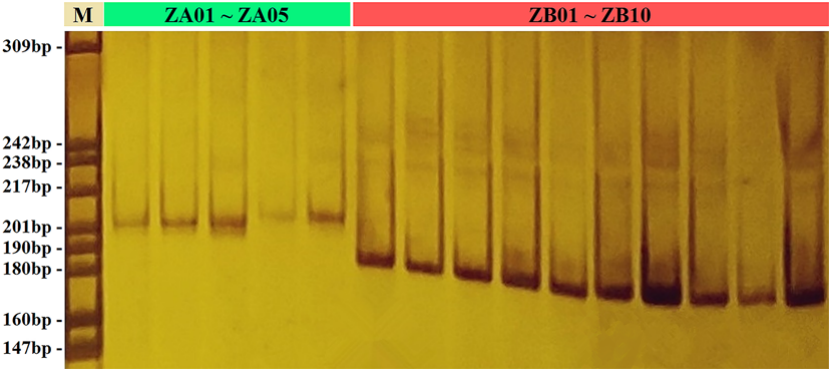
Polymorphisms revealed by ZBg46 in 15 individuals of *Zanthoxylum* In the figure, the marker (M) was pBR322 DNA/*M*spI, the amplified bands from left to right were ZA01 to ZA05 (under the green stripe) and ZB01 to ZB10 (under the red stripe).

To assess the degree of polymorphism for potential SSR markers, 100 primer pairs were selected randomly and validated across 15 individuals (ten ZB and five ZA individuals). Mononucleotide SSRs were not considered. PCR amplification showed that 85 primer pairs produced fragments that were clear and stable. Of these, 36 SSR loci were polymorphic after amplification products were separated (Table 6 and Figure 3). The polymorphism rate (polymorphic markers/number of markers used for polymorphic screening; 36/85) of the G-SSR markers tested is higher than that of EST-SSR markers developed in previous studies (18/55 and 15/44) [34,35]. One reason may be that exon sequences are more conserved than intron or intergenic sequences [36]; this suggests that in conserved regions, the relatively low frequency of polymorphisms may limit the utility of EST-SSR markers; consequently, the development of G-SSR markers is necessary [37,38].

**Table 6:** Characterizations of 36 polymorphic G-SSR primers pairs

Primer name	Primer seqence (5′-3′)	Repeat motif	Expected size (bp)	All individuals				*Z. bungeanum*				*Z. armatum*			
				Na	PIC	Ho	He	Na	PIC	Ho	He	Na	PIC	Ho	He
ZBg01	F: TGTCTTCGCCTTCCATTCTC	(AG)10	272	3	0.48	0.13	0.57	2	0.16	0.20	0.19	2	0.27	0.00	0.36
	R: CGAGCACCAACCCTAACAAT														
ZBg02	F: GGGTGAGACTGGCGTTATGT	(TA)8	268	3	0.58	0.40	0.68	2	0.36	0.60	0.51	1	0.00	0.00	0.00
	R: CGAACCAAGTCATTGAGGCT														
ZBg03	F: GCTTCGTCAGGCAGAAACTC	(AG)8	238	4	0.59	0.64	0.68	3	0.51	0.70	0.62	1	0.00	0.00	0.00
	R: CAAAATCGGTCTTCGCTTTC														
ZBg04	F: GATGCGACCCTCACTCTAGC	(AGA)9	180	5	0.69	0.36	0.77	4	0.65	0.17	0.77	5	0.72	0.60	0.84
	R: GTCGTCCGAATTGGAGGTAA														
ZBg07	F: TCACTCCTATGCCTCCTTGG	(TA)10	219	5	0.58	0.40	0.64	3	0.30	0.10	0.35	5	0.70	1.00	0.82
	R: TGATCTTGGTGCCACAGGTA														
ZBg11	F: CATTGGCACACCAAGTGTTT	(TA)8	248	3	0.47	0.11	0.57	3	0.50	0.13	0.61	1	0.00	0.00	0.00
	R: TCGAATTTTAGCACTGCTCG														
ZBg17	F: GGCAATCTTCTCACCATTCC	(AG)13	275	2	0.27	0.40	0.34	2	0.27	0.40	0.34	–	–	–	–
	R: TGAGGTGGATGCACATAAGG														
ZBg18	F: GCCCAGTTGTCAGTTTTGGT	(GT)10	246	5	0.66	0.00	0.73	3	0.47	0.00	0.57	3	0.55	0.00	0.71
	R: ATGGGCATGAGATGGCTTAG														
ZBg19	F: TGGATTCACTCATTCACATGC	(TAT)8	206	2	0.33	0.47	0.43	2	0.22	0.30	0.27	2	0.36	0.80	0.53
	R: TTTGGAGTTCAAACCTCGCT														
ZBg21	F: GGCCGCCTGAAGAATACAT	(ATT)8	142	3	0.30	0.38	0.34	2	0.19	0.25	0.23	2	0.33	0.60	0.47
	R: TTCGGCTAACCAAACAAACC														
ZBg24	F: AACGCGCCATTTCATATTTC	(AT)8	189	5	0.73	0.54	0.80	4	0.66	0.50	0.76	2	0.33	0.60	0.47
	R: AGAGCATTGAGCCTCGTTGT														
ZBg30	F: CCCATGAGAGTTGACTGCAA	(TTTA)5	230	4	0.60	0.86	0.68	3	0.54	1.00	0.65	2	0.33	0.60	0.47
	R: CAGTGCCTGACAGAGTCGAG														
ZBg31	F: GTACAAGCGATGCGACAGAA	(TA)14	256	2	0.36	0.00	0.49	1	0.00	0.00	0.00	1	0.00	0.00	0.00
	R: AGTGCGTGACTCGAACAGTG														
ZBg34	F: CCAACATCAAAGAAACGCAA	(AAT)6	163	2	0.37	0.00	0.51	2	0.33	0.00	0.44	1	0.00	0.00	0.00
	R: CATAATTCCTAGGTTGGCCG														
ZBg35	F: GCTGTGAACATGAAATCGGA	(TA)13	189	3	0.40	0.33	0.46	3	0.40	0.33	0.46	–	–	–	–
	R: TCGCGTGAAATAGAATGTCG														
ZBg36	F: TGGCATGTTTGGTTCTCTTG	(AG)10	271	2	0.16	0.20	0.19	2	0.22	0.30	0.27	1	0.00	0.00	0.00
	R: AGAAGACCTGGGTGTGGTTG														
ZBg40	F: GTCGTCAAATGAACCGTGTG	(TATATG)5	204	4	0.50	0.40	0.61	4	0.44	0.60	0.50	1	0.00	0.00	0.00
	R: AATCGATTCGGTGTGTGGAT														
ZBg45	F: ACGTGATTGGTAGGAGACGG	(AT)12	272	3	0.47	0.56	0.57	3	0.47	0.56	0.57	–	–	–	–
	R: ATGGGTCCACGGGTACATAA														
ZBg46	F: AATCCTTCCCCATCTCAAGC	(TAT)7	173	3	0.44	0.00	0.51	1	0.00	0.00	0.00	2	0.36	0.00	0.53
	R: CCCGATATTTTCCCAATGTG														
ZBg71	F: GAATATGGGGAAGGAAACCAA	(TATG)5	204	5	0.45	0.33	0.49	4	0.39	0.13	0.44	3	0.47	0.75	0.61
	R: TATTATGAATGGCGTGGGGT														
ZBg73	F: GGATGCCAATCCTTCACACT	(ATT)9	263	3	0.59	0.23	0.69	2	0.36	0.00	0.50	2	0.33	0.60	0.47
	R: TGAATAGTACTTGGGGGCCA														
ZBg74	F: TCCACGTCAACTCCAAACAA	(AT)9	259	3	0.49	0.07	0.60	2	0.24	0.11	0.29	1	0.00	0.00	0.00
	R: GACTCAACTGTCGGTGCTCA														
ZBg76	F: ACATCTCCGGTCGATCTTGT	(TA)12	270	3	0.47	0.00	0.57	2	0.21	0.00	0.26	1	0.00	0.00	0.00
	R: ATTGGAGATCGAGGAACACG														
ZBg77	F: CCATCATCTTCCATGATTGCT	(TTC)6	277	4	0.57	0.00	0.64	2	0.27	0.00	0.34	2	0.27	0.00	0.36
	R: GGTCTTCCAAATTCGAACCA														
ZBg83	F: GGGTTCTACCCTAGCCGAAC	(AT)12	266	4	0.46	0.23	0.53	1	0.00	0.00	0.00	4	0.54	0.60	0.64
	R: GGTTCCGATTTCAGTTCCAA														
ZBg84	F: ACGATATGAAACGGAAACGG	(AAG)11	225	5	0.67	0.00	0.74	3	0.47	0.00	0.57	2	0.35	0.00	0.53
	R: GATTCCAAGAAATGCCTCCA														
ZBg86	F: AGTTGGAATGAGAACATGGACA	(TAT)6	209	2	0.27	0.27	0.33	1	0.00	0.00	0.00	2	0.36	0.80	0.53
	R: TGCGACGCTATCACAAACTT														
ZBg89	F: GAGCCTAGAACAGCGTCGTC	(TATG)8	229	3	0.35	0.33	0.40	2	0.27	0.20	0.34	2	0.33	0.60	0.47
	R: AAACCTGAAAGGCAGCTTGA														
ZBg90	F: CATTTTGTGCGATAGGCAGA	(TAT)11	242	2	0.34	0.09	0.45	2	0.26	0.13	0.33	2	0.35	0.00	0.53
	R: CTAGGAGACAGCCCAGCAAC														
ZBg91	F: CCATGCAACAGCGATTCTAA	(TG)9	262	5	0.47	0.43	0.52	3	0.19	0.22	0.22	3	0.59	0.80	0.73
	R: TCCACACACATGTCAAACACA														
ZBg92	F: CGCTGCCATTATTTGCTGTA	(ATA)12	249	7	0.75	0.73	0.81	4	0.58	0.60	0.68	4	0.60	1.00	0.73
	R: TGGTGGCACTTAGCAGTGAG														
ZBg94	F: TAATACTCGGCCATGAACCC	(AAAAT)5	202	3	0.40	0.15	0.48	3	0.34	0.22	0.39	2	0.38	0.00	0.57
	R: CGAATGACGTGGTGAAGAAG														
ZBg95	F: CAGGATCGACCTCCACAGTT	(TTA)11	279	3	0.55	0.67	0.65	3	0.59	0.78	0.70	2	0.24	0.33	0.33
	R: AATGTCGCCAAAGTAGCGTC														
ZBg96	F: AATATTGTTTGGGGGCCATT	(GAA)7	279	5	0.60	0.00	0.68	3	0.49	0.00	0.61	3	0.50	0.00	0.62
	R: TTTATGGATGCCAAGCCTTC														
ZBg97	F: CATAGCACAAGCAATGTGGG	(TA)10	162	3	0.42	0.07	0.48	1	0.00	0.00	0.00	3	0.49	0.20	0.64
	R: ACACCTCCAGACCAGTCCAC														
ZBg98	F: TGGAATGAGGTCTTCCAAGG	(TTC)6	190	3	0.35	0.20	0.40	2	0.16	0.00	0.19	3	0.55	0.60	0.69
	R: ATGACAAGCTTTCGGCAGTT														

Abbreviations: He, expected heterozygosity; Ho, observed heterozygosity; Na, observed number of alleles; PIC, polymorphism information content.

In total, 126 alleles with a range of 2 to 7 per loci (mean = 3.5) were obtained from the 36 polymorphic SSR loci. The PIC, Ho, and He values per locus ranges from 0.16 to 0.75 (mean = 0.48), 0.00 to 0.86 (mean = 0.28), and 0.19 to 0.81 (mean = 0.56), respectively. According to the classification criteria of Bostein et al. [39], loci polymorphisms can be divided into three degrees: low (PIC < 0.25), moderate (0.25 < PIC < 0.5), and high (PIC > 0.5). Among the 36 polymorphic G-SSR markers, 1 (2.78%), 22 (61.11%), and 13 (36.11%) were demonstrated to have low, moderate, and high polymorphism, respectively, in 15 individuals. Because these SSR markers were developed based on a single ZB genome sequence, three markers (ZBg17, ZBg35 and ZBg45) did not amplify any fragment, and nine markers (ZBg02, ZBg03, ZBg11, ZBg31, ZBg34, ZBg36, ZBg40, ZBg74 and ZBg76) amplified only one fragment (i.e., were not polymorphic) in the five ZA individuals. Interestingly, four markers (ZBg46, ZBg83, ZBg86, and ZBg97) that are not polymorphic in ZB are polymorphic in ZA, and three markers (ZBg04, ZBg07 and ZBg98) produce more alleles in ZA than ZB, suggesting that some loci might be more likely to mutate in ZA relative to ZB. Similar results have been reported for *Vernicia fordii* [40], *Taxus wallichiana* [41], and *Saxifraga sinomontana* [42].

**Figure 4:**
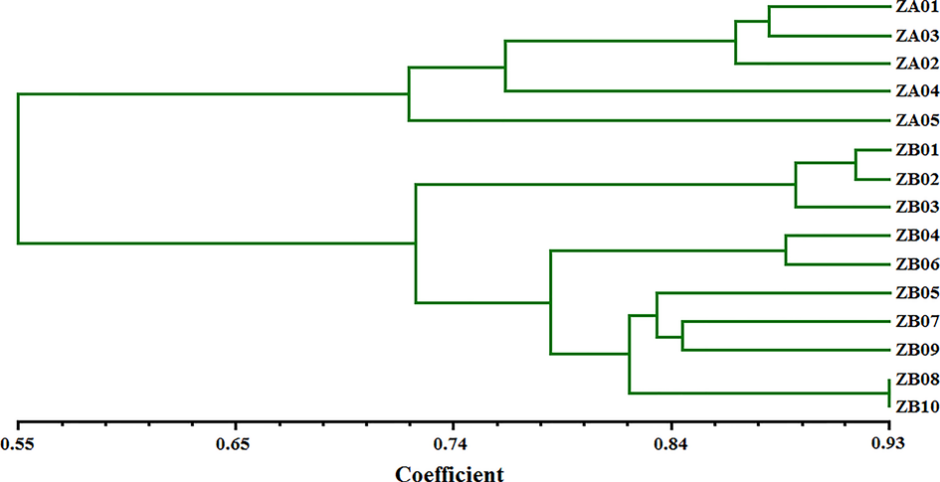
Cluster diagram for 15 individuals of *Zanthoxylum* by UPGMA method

Based on the 36 polymorphic SSR markers identified, the genetic relationships among the 10 ZB individuals and 5 ZA individuals were investigated using UPGMA clustering. The dendrogram shows that the genetic similarity coefficient (GSC) ranges from 0.55 to 0.93 and the 15 individuals are distributed into two major clusters by species (Cluster I: ZA; Cluster II: ZB) (Figure 4). When the genetic similarity coefficient (GSC) value is approximately 0.72, Cluster I and Cluster II each divide into two subclusters: I-A, I-B, II-A, and II-B. Cluster I-A includes four individuals (ZA01-ZA04) that are from adjacent provinces (Sichuan and Chongqing), and Cluster I-B includes ZA05 (Goujiao). In Cluster II-A, the three individuals (ZB01-ZB03) are from adjacent provinces (Sichuan and Guizhou). However, Cluster II-B consists of seven individuals (ZB04-ZB10) that are from three provinces (Shaanxi, Shanxi, and Shandong). Hancheng of Shaanxi is one of the main producing areas of ZB. Therefore, we speculate that ZB05 and ZB07 were probably introduced from Hancheng. In addition, although 36 G-SSR markers were used, ZA08 and ZA10, which came from the same area with different name, are indistinguishable from each other, suggesting that they came from the same individual. The clusters support the expected classification of ZA and ZB and demonstrate the efficacy of the G-SSR markers developed in the present study.

## Conclusions

In the present study, genomic characteristics of ZB were obtained by a genome survey, and G-SSRs were identified and developed simultaneously from the sequence data. The results showed that ZB has a notably complex genome. Its genome size is 3971.92 Mb, with a heterozygosity rate, repeat sequence rate, and GC content of 1.73%, 86.04%, and 37.21%, respectively. For future whole-genome sequencing, we propose that using Illumina and PacBio sequencing technologies combined with Hi-C and BioNano for assist will yield better genome assembly results. A total of 27153 G-SSRs were identified, and 21243 non-redundant primers pairs were designed. Thirty-six of one hundred randomly selected primer pairs showed polymorphism among ten ZB individuals and five ZA individuals. An UPGMA-derived dendrogram showed that the clustering of these 15 individuals was consistent with their species of origin. These findings will be useful for future genomic and genetic studies in ZB.

## Supplementary Material

Supplementary Table S1

## Abbreviations

EST: expressed sequence tag
GSC: genetic similarity coefficient
He: expected heterozygosity
Hi-C: high-through chromosome conformation capture
Ho: observed heterozygosity
*K*-mer: a sequence of k characters in a string
Na: number of alleles
NGS: next-generation sequencing
PIC: polymorphism information content
SSR: simple sequence repeat
STR: short tandem repeats
UPGMA: unweighted pair group method with arithmatic mean
ZA: *Zanthoxylum armatum*
ZB: *Zanthoxylum bungeanum*
